# Supplementary material for: Real-World Implementation of PRISMA-7 and Clinical Frailty Scale for Frailty Identification and Integrated Care Activation: A Cross-Sectional Study in Northern Italian Primary Practice
Source: J Clin Med. 2025 May 14;14(10):3431. doi: 10.3390/jcm14103431 (PMC12111854; doi:10.3390/jcm14103431)
Supplement: Supplementary file 1 [file jcm-14-03431-s001.zip › jcm-3621949-supplementary.pdf]

Supplementary Material

Tables and Figures

Table S1. Missing data overview for PRISMA-7 and Clinical Frailty Scale (CFS) screening results.

Participating patients (n=19,501)	PRISMA-7-score	CFS-score	n (%)
Overall missing values			
Completely missing	missing	missing	829 (4.3)
Only PRISMA-7 missing	missing	not missing	14 (0.1)
PRISMA-7-positive, CFS missing	3–7	missing	612 (3.1)
n total missing			1,455 (7.5)
Analyses using PRISMA-7 cut-off 3			
Excluded: PRISMA-7-negative, CFS positive	0–2	5–9	42 (0.2)
n total included in the analysis			18,004
Analyses using PRISMA-7 cut-off 4			
Excluded: PRISMA-7-negative, CFS positive	0–3	5–9	376 (1.9)
n total included in the analysis			17,670

Abbreviation: CFS Clinical Frailty Scale.

Table S2. Comparison of demographic characteristics between participating and non-participating general practitioners (GPs).

Variable	Participating GPs	Non-participating GPs	*p*-value
Age, median (IQR)	47 (39–58)	58 (45.5–64)	< 0.001[1]
Sex			0.079[2]
Female	72 (50.7)	61 (39.9)	
Male	70 (49.3)	92 (60.1)	
Location of the GP office			0.007[2]
Urban area	75 (52.8)	56 (36.6)	
Rural area	67 (47.2)	97 (63.4)	

[1] Mann-Whitney-U test. [2] Fisher's exact U test. Abbreviations: GPs General practitioners, IQR Interquartile range.

Table S3. Frailty prevalence according to age group, sex, and GP office location, using PRISMA-7 (cut-offs ≥3 and ≥4) and CFS (score ≥5).

Subgroup	PRISMA-7 ≥3	PRISMA-7 ≥4	CFS ≥5
Age 75–84 years	4,027 / 13,113 (30.7%)	2,048 / 13,113 (15.6%)	1,468 / 3,725 (39.4%)
Age ≥85 years	4,555 / 5,545 (82.1%)	3,324 / 5,545 (59.9%)	2,384 / 4,245 (56.2%)
Male	4,102 / 7,966 (51.5%)	2,336 / 7,966 (29.3%)	1,469 / 3,776 (38.9%)
Female	4,479 / 10,691 (41.9%)	3,035 / 10,691 (28.4%)	2,382 / 4,193 (56.8%)
Urban GP office	4,994 / 11,181 (44.7%)	3,192 / 11,181 (28.5%)	2,340 / 4,618 (50.7%)
Rural GP office	3,588 / 7,477 (48.0%)	2,180 / 7,477 (29.2%)	1,512 / 3,352 (45.1%)

Proportions are given as number of frail patients / total in subgroup (%). Abbreviations: GP General Practitioner; CFS Clinical Frailty Scale.

Table S4. Frailty classification combinations by subgroup: Comparison of PRISMA-7 (cut-off 3 and 4) with CFS.
A. Classification According to PRISMA-7 Cut-off ≥3 (n = 18,004).

Subgroup	Not Frail (PRISMA−)	Frail PRISMA+ / CFS−	Frail PRISMA+ / CFS+	p-value
Age 75–84	9,047 (70.8%)	2,257 (17.7%)	1,468 (11.5%)	<0.001
Age ≥85	987 (18.9%)	1,861 (35.6%)	2,384 (45.6%)	
Male	3,849 (50.5%)	2,307 (30.3%)	1,469 (19.3%)	<0.001
Female	6,185 (59.6%)	1,811 (17.5%)	2,382 (23.0%)	
Urban GP	6,172 (57.2%)	2,278 (21.1%)	2,340 (21.7%)	<0.001
Rural GP	3,862 (53.5%)	1,840 (25.5%)	1,512 (21.0%)	

B. Classification According to PRISMA-7 Cut-off ≥4 (n = 17,670)

Subgroup	Not Frail (PRISMA−)[1]	Frail PRISMA+ / CFS−	Frail PRISMA+ / CFS+	p-value[2]
Age 75–84	10,811 (84.7%)	703 (5.5%)	1,253 (9.8%)	<0.001
Age ≥85	2,099 (39.6%)	937 (17.7%)	2,265 (42.7%)	
Male	5,510 (71.2%)	867 (11.2%)	1,364 (17.6%)	<0.001
Female	7,400 (71.7%)	773 (7.5%)	2,153 (20.9%)	
Urban GP	7,771 (71.8%)	919 (8.5%)	2,137 (19.7%)	0.003
Rural GP	5,139 (71.0%)	721 (10.0%)	1,381 (19.1%)	

[1] PRISMA-7 score below cut-off; CFS not assessed. [2] p-values calculated using Chi² test. Abbreviations: CFS Clinical Frailty Scale; GP General Practitioner.

Table S5. Extended logistic regression models comparing combined frailty status groups (PRISMA-7 and CFS).

A. Frail by both tools versus non-frail.

Predictor	PRISMA-7 ≥3	PRISMA-7 ≥4
n	13,885	16,029
Nagelkerke R^2	0.400	0.328
Age (per year)	1.34 (1.32–1.35)***	1.28 (1.27–1.30)***
Female sex	0.75 (0.69–0.83)***	n.s.
Rural GP office	n.s.	n.s.
Constant	−24.63***	−22.06***

B. Frail by both tools vs. PRISMA+/CFS−.

Predictor	PRISMA-7 ≥3	PRISMA-7 ≥4
n	7,969	5,157
Nagelkerke R^2	0.109	0.046
Age (per year)	1.09 (1.08–1.10)***	1.05 (1.04–1.06)***
Female sex	1.88 (1.72–2.07)***	1.67 (1.48–1.88)***
Rural GP office	0.83 (0.76–0.91)***	n.s.
Constant	−7.80***	−3.89***

C. PRISMA+/CFS− vs. PRISMA−.

Predictor	PRISMA-7 ≥3	PRISMA-7 ≥4
n	14,152	14,152
Nagelkerke R^2	0.245	0.189
Age (per year)	1.24 (1.23–1.25)***	1.23 (1.22–1.25)***
Female sex	0.42 (0.38–0.45)***	0.60 (0.53–0.67)***
Rural GP office	1.32 (1.22–1.43)***	n.s.
Constant	−17.86***	−19.15***

Abbreviations: CFS Clinical Frailty Scale; GP General Practitioner; OR Odds Ratio; CI Confidence Interval; n.s. not significant. *** $p < 0.001$

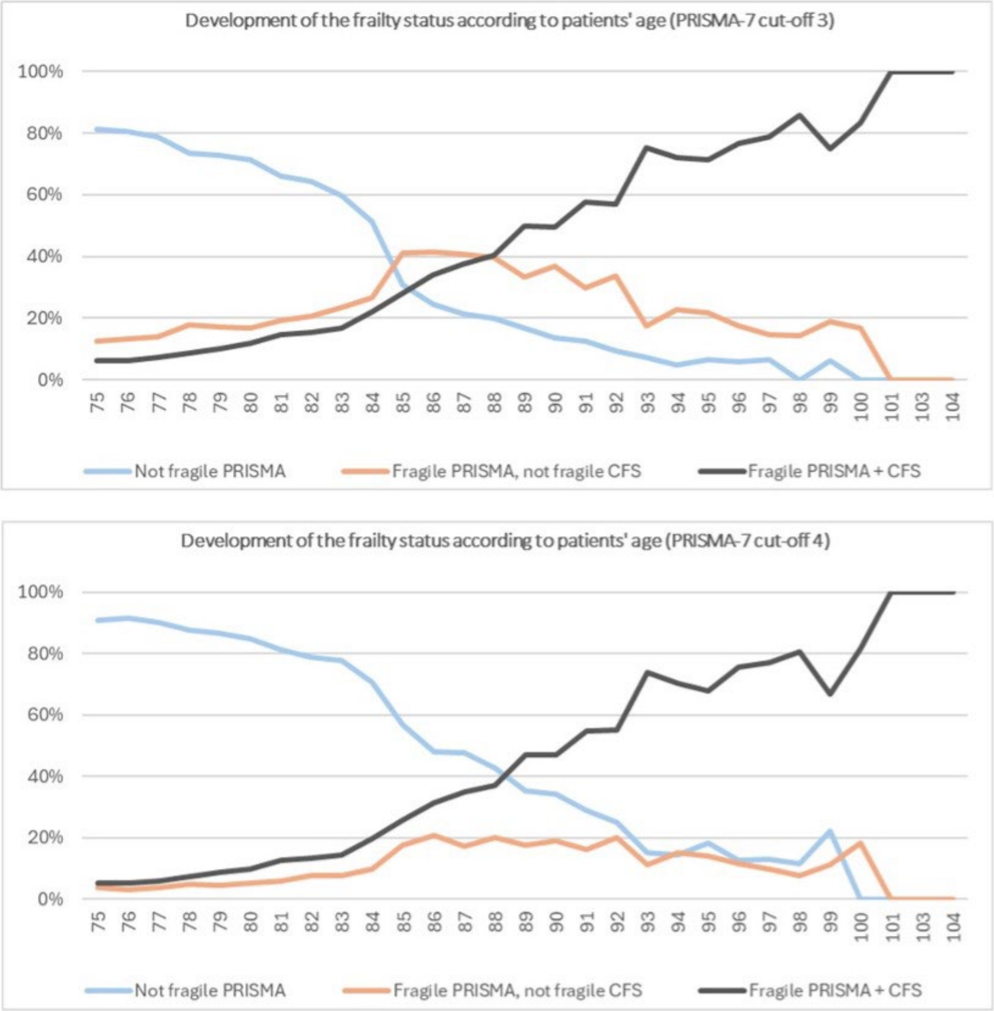

Figure S1. Evolution of frailty status by age and PRISMA-7 cut-off. Line charts depicting the percentage distribution of three frailty classification groups by patient age (in years), using PRISMA-7 cut-off 3 (top panel) and cut-off 4 (bottom panel). The groups shown are: Not frail (PRISMA-7 score below cut-off); frail according to PRISMA-7 only (CFS-negative); frail according to both PRISMA-7 and CFS (CFS score ≥5). Frailty prevalence increased steeply with age across both cut-offs. The group "frail by both tools" became dominant from age 85 onward. At cut-off 3, the proportion of discordant cases (PRISMA+ / CFS−) peaked around age 87 and declined in older ages. Cut-off 4 markedly reduced this discordant group and shifted the age-related trajectory of frailty identification. Abbreviations: CFS Clinical Frailty Scale.

Figure S3. Percentage of frail patients per year-of-age by tool (PRISMA-7 cut-off 3 and 4, CFS), stratified by sex.

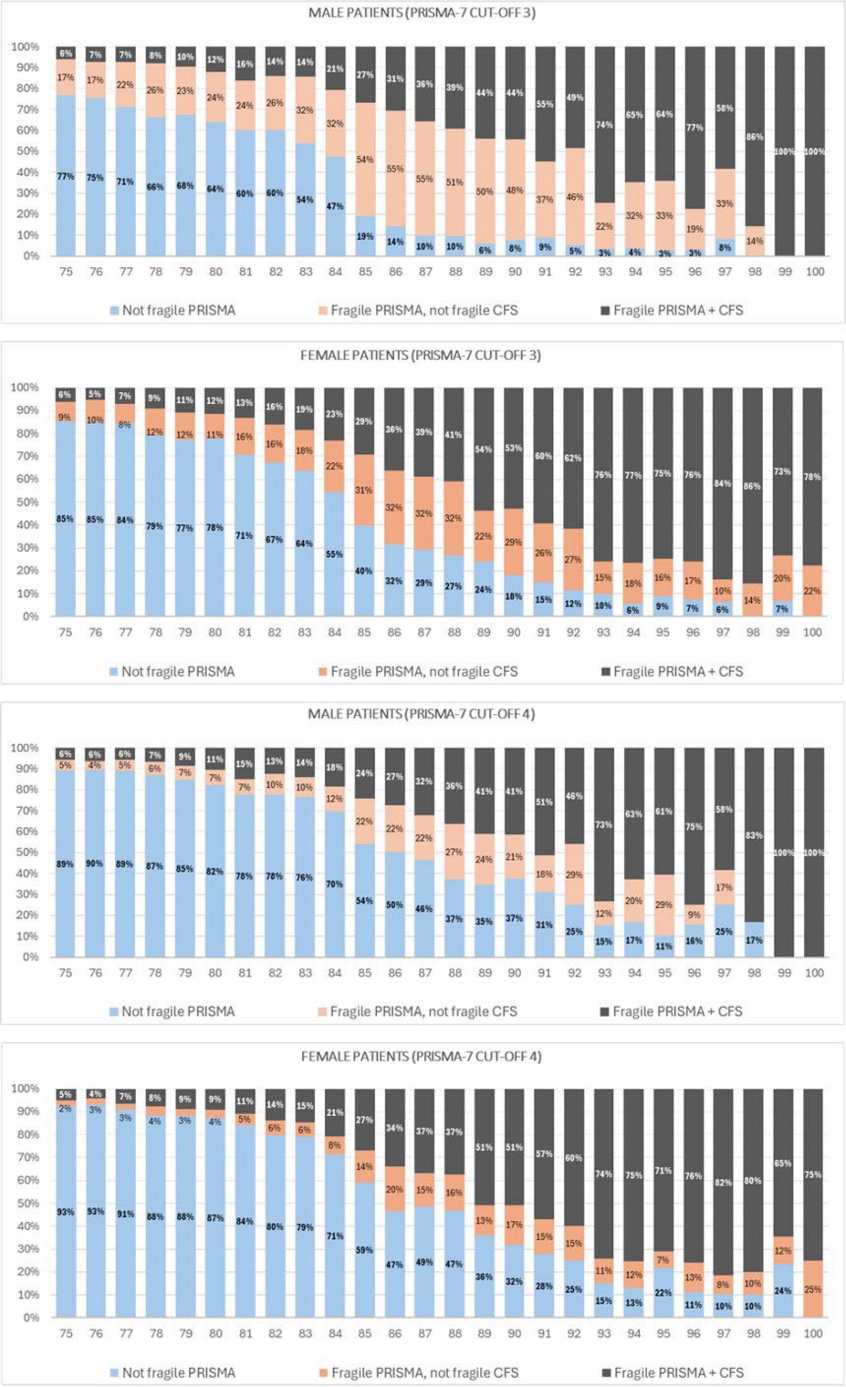

Figure S2. Sex-specific frailty classification by age and PRISMA-7 cut-off. Stacked bar charts showing the distribution of frailty classification categories by exact age (in years) and sex, stratified for PRISMA-7 cut-off 3 (upper panels) and cut-off 4 (lower panels). Frailty classifications are grouped into: Not frail according to PRISMA-7 (light blue); frail according to PRISMA-7 but not CFS (orange); frail according to both PRISMA-7 and CFS (black). Among both sexes, the proportion of patients classified as “not frail” decreased with increasing age, while the proportion “frail by both tools” increased steadily. Males showed higher discordance (PRISMA+ / CFS−) across most ages, particularly with cut-off 3. With cut-off 4, this discordant group diminished in both sexes, leading to a more consistent increase in dual frailty classification with age. Abbreviations: CFS Clinical Frailty Scale.

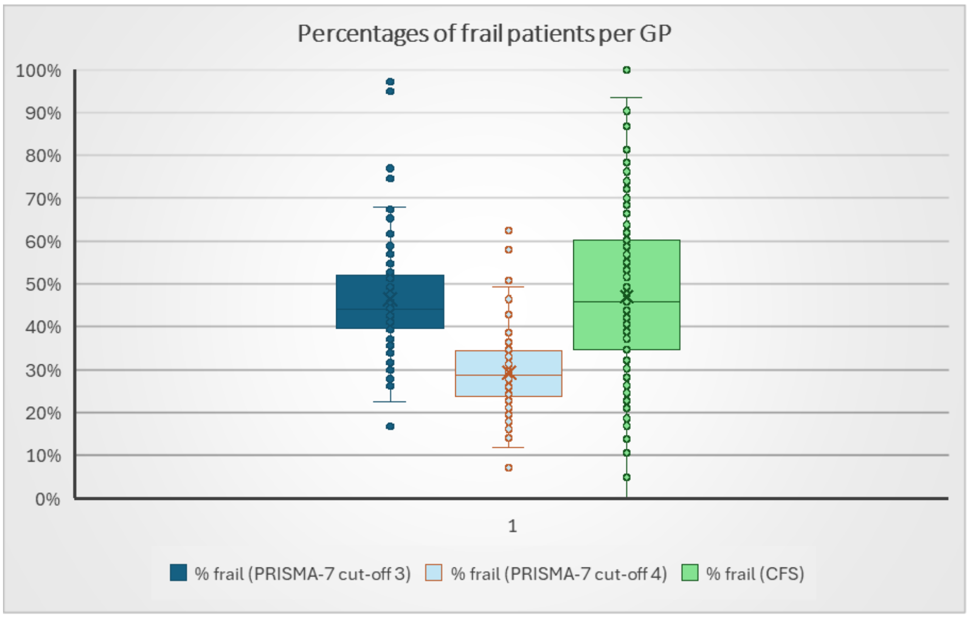

Figure S3. Inter-GP variability in frailty classification using PRISMA-7 and CFS. Boxplots showing the distribution of frailty prevalence per general practitioner (GP), expressed as the percentage of assessed patients classified as frail. Frailty was assessed using PRISMA-7 (cut-off ≥3 and ≥4) and the Clinical Frailty Scale (CFS, score ≥5). Each point represents one GP. The median percentage of frail patients per GP was 44% (IQR 40–52%) with PRISMA-7 cut-off 3, 29% (IQR 24–34%) with cut-off 4, and 46% (IQR 35–60%) for CFS. A few GPs classified nearly all patients as frail; two outlier GPs scored 100% of their PRISMA-positive patients as frail on CFS. These results highlight marked heterogeneity in frailty assessment and classification at the provider level.
Abbreviations: GP General Practitioner; CFS Clinical Frailty Scale.
